# Chemical fluorophores for fluorescence lifetime imaging

**DOI:** 10.1039/d5cs00280j

**Published:** 2026-01-07

**Authors:** Clarissa Lim, Deborah Seah, Marc Vendrell

**Affiliations:** a School of Chemistry, Chemical Engineering and Biotechnology, Nanyang Technological University Singapore 637371 Singapore; b Centre for Inflammation Research, Institute for Regeneration and Repair, The University of Edinburgh Edinburgh EH16 4UU UK marc.vendrell@ed.ac.uk; c IRR Chemistry Hub, Institute for Regeneration and Repair, The University of Edinburgh Edinburgh EH16 4UU UK

## Abstract

Fluorescence lifetime imaging has emerged as a promising modality to extract molecular information from biological systems, providing detailed and semi-quantitative characterisation of subcellular microenvironments. Fluorescence lifetime measurements offer robust insights into biological processes as they are less dependent on concentration and excitation power than intensity-based measurements. However, fluorescence lifetime imaging suffers from a paucity of fluorophores with wide dynamic ranges of lifetimes and responsiveness to biostimuli. This shortcoming has prompted the design of new chemical strategies to tailor the optical properties of organic fluorophores and their application in multiplexed live-cell imaging for the visualisation of molecular and cellular interactions. This Review article covers advances – primarily from the last 5 years – in the chemical design of fluorescence lifetime probes that combine optical reporters and targeting groups (*e.g.*, ligands, peptides, proteins), their applications in bioimaging and related computational-based innovations for data acquisition and analysis. The perspectives and challenges in the design and applications of fluorescence lifetime probes are discussed, bridging chemistry and bioimaging as well as providing strategic insights for advancing fluorescence lifetime imaging.

## Abstract 2

Fluorescence lifetime imaging has emerged as a promising modality to extract molecular information from biological systems, providing detailed and semi-quantitative characterisation of subcellular microenvironments.

## Graphical abstract

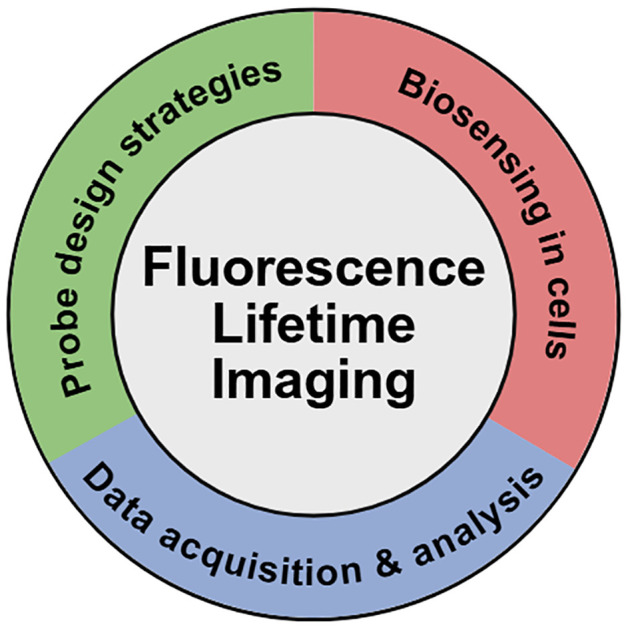

**Key learning points**

(1) The fluorescence lifetimes of organic fluorophores can be modulated by derivatisation of chemical structures, especially those affecting photoinduced electron transfer (PeT) or intramolecular charge transfer (ICT).

(2) Fluorescence lifetime probes provide valuable information about subcellular microenvironments.

(3) Fluorescence lifetime imaging microscopy (FLIM) can differentiate healthy and diseased states by tracking endogenous biomolecules or exogenous targeted fluorophores.

(4) Extracting biological data from FLIM experiments requires balancing photon budget, accuracy of fluorescence decay and spatiotemporal resolution.

(5) Perspectives and future challenges in FLIM.

## Introduction

Fluorophores have become indispensable chemical tools for visualizing, interrogating and understanding biological processes in cells, tissues and complex organisms.^1–8^ Fluorescent probes can be utilised in a broad range of mechanistic studies, from those monitoring protein expression and/or enzymatic activity in cells to real-time analyses of drug trafficking and cell-to-cell interactions.^9–15^ Among their main features, fluorophores stand out because of their (1) chemical tunability which make them compatible with many molecular and cellular targets, (2) relatively low cost compared to radiolabeled imaging agents, and (3) multiplexed capabilities for simultaneous imaging of different biomarkers.^16^

The detailed characterisation of complex biological processes in cells and tissues often requires concurrent tracking of multiple targets. To date, most multi-color fluorescence imaging studies have relied on spectrally resolved intensity measurements, with typically an average of 4 to 5 fluorophores being detected within the UV-to-NIR spectral region.^17,18^ In contrast to spectrally-resolved microscopy, time-resolved fluorescence microscopy measures the rate of excited-state decay to determine fluorescence lifetime, an intrinsic property that is largely independent of concentration and excitation power.^19^ As a result, lifetime measurements potentially enhance consistency and reproducibility compared to intensity-based measurements. Furthermore, their sensitivity to small variations in local environments (*e.g.*, pH, viscosity, polarity) has created new avenues in the development of smart fluorescent probes with specific and distinguishable readouts under different subcellular microenvironments.^20,21^

Fluorescence lifetime imaging microscopy (FLIM) leverages the advantages of lifetime measurements by converting lifetime information into spatial contrast, providing a more robust imaging modality alternative to intensity-based imaging. By exploiting the environmental sensitivity of fluorescence lifetimes, FLIM can provide quantitative insights into the fluorophore microenvironment beyond localisation and intensity.^22,23^ To maximise the performance of such fluorophores in biological systems, there is a need to optimise chemical structures that can generate photophysical diversity as well as achieve target specificity.

**Fig. 1:**
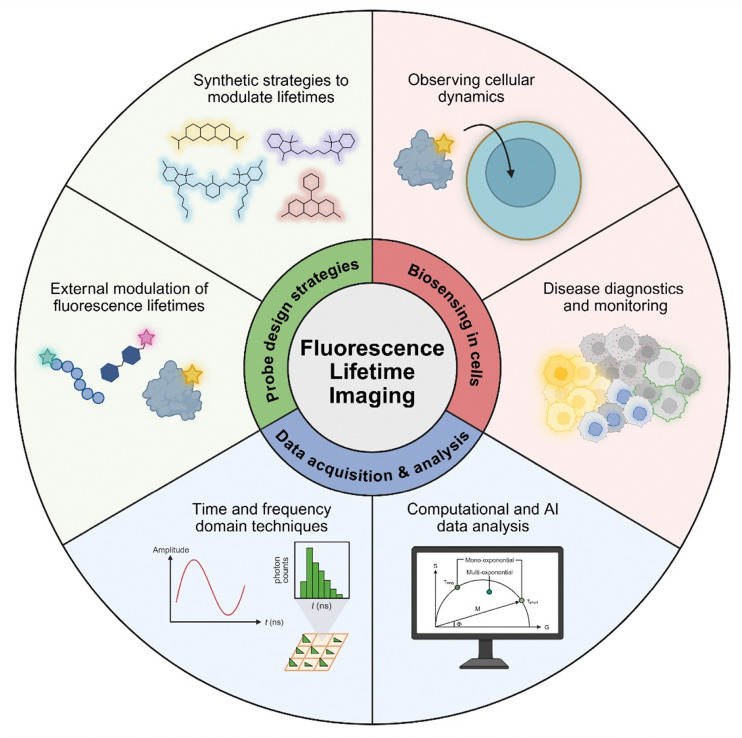
Schematic illustration of the main research areas in fluorescence lifetime imaging.

In this Review, we will present the latest advances – primarily from the last 5 years – in the chemical design and applications of fluorescence lifetime imaging probes (Fig. 1). With a few exceptions, our article does not discuss Förster resonance energy transfer (FRET) probes or genetically-encoded fluorescent proteins^24,25^ because these have been reviewed elsewhere.^26–31^ Our Review focuses on the chemical designs of organic fluorescent probes that combine optical reporters (*e.g.*, small molecule fluorophores) and targeting units (*e.g.*, molecular ligands, peptides, proteins). This Review discusses the various synthetic strategies to modulate lifetimes for different classes of fluorophores and includes examples on how these probes can be used for bioimaging purposes, with fluorescence lifetime as a quantitative readout for cellular processes. Our Review also discusses strategies for FLIM data acquisition and computational approaches for lifetime data analysis in biological models, providing insight on how emerging techniques can be used to accelerate the application of FLIM in biological studies.

## Chemical strategies for fluorescence lifetime modulation

Fluorescence lifetime (*τ*) is defined as the average time in which an excited fluorophore remains in the excited state before returning to the ground state.^32^ Control over fluorescence lifetime is crucial for the development of suitable fluorophores for time-resolved fluorescence microscopy, particularly for multiplexed imaging of dynamic biological processes. Fluorophores for confocal imaging should ideally be photostable, compatible with available excitation sources and sufficiently bright (*i.e.*, high quantum yields and extinction coefficients) to reduce imaging time. For FLIM experiments in particular, fluorophores should possess (1) high sensitivity to environmental changes reported by large variations in their lifetimes and (2) large dynamic ranges (*i.e.*, the range in which fluorescence lifetime varies in response to an external stimulus) to maximise the combination of fluorophores with different readouts.^33^

To tune lifetime-relevant properties, it is essential to consider the determinants of fluorescence lifetime, namely the radiative (*k*_r_) and non-radiative (*k*_nr_) rate constants, and, by extension, fluorescence quantum yields. *k*_r_ is an intrinsic rate dependent on the oscillator strength of the fluorophore; therefore, it is weakly influenced by environmental effects. Conversely, *k*_nr_ can vary because of both intrinsic and extrinsic factors such as intersystem crossing to the excited triplet state, vibrational relaxation and energy transfer. Microenvironmental sensitivity arises from these non-radiative processes and therefore the lifetimes of some fluorophores vary in a microenvironment-dependent manner, as will be discussed in subsequent sections. Given that non-radiative processes play an important role in determining fluorescence lifetimes, precise control over such mechanisms opens avenues for lifetime modulation. In this section, we will discuss how different synthetic strategies and environmentally responsive elements have been employed to modulate photophysical characteristics, including the dynamic range and environmental sensitivity of fluorescence lifetime, in different fluorophore scaffolds.

### Synthetic control of fluorescence lifetimes in fluorophores

Synthetic approaches for lifetime modulation rely on structural modification of a fluorophore to regulate the excited-state energy transfer processes governing *k*_nr_.^34^ As a result, the introduction of chemical groups that inhibit or promote such processes can be leveraged to precisely tune the observed lifetime. The main mechanisms include (1) photoinduced electron transfer (PeT), where electron transfer between a donor or acceptor moiety (either within the fluorophore or involving an external quencher) leads to fluorescence quenching and shorter lifetimes, (2) intramolecular charge transfer (ICT), in which electronic redistribution from an electron-rich donor to an electron-deficient acceptor stabilizes the excited state to alter lifetimes, and (3) twisted intramolecular charge transfer (TICT), as a subtype of ICT where structural twisting of the donor–acceptor pair in the excited state enhances non-radiative pathways, resulting in shorter lifetimes. These mechanisms form the foundation for designing organic fluorophores with tailored lifetime properties for FLIM applications.

**Fig. 2:**
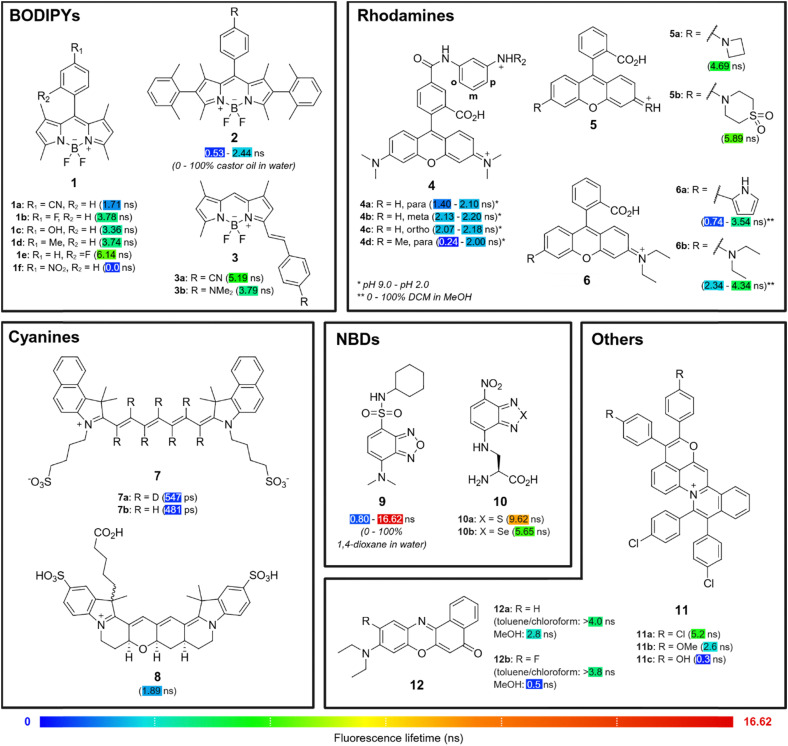
Lifetime fluorophores. Representative examples of the diversification of organic fluorophores to produce fluorescence lifetime probes.

4,4-Difluoro-4-bora-3*a*,4*a*-diaza-*s*-indacenes (BODIPYs) are synthetically versatile fluorophores with high fluorescence quantum yields, good photostability and biocompatibility, making them attractive candidates for biological imaging.^35^ Modifications at the *meso* position are well studied for their ability to induce PeT, which results in shorter fluorescence lifetimes in the fluorophore.^36^ Aromatic substituents at this site, typically benzene derivatives, can be functionalised with groups that confer different electronic and steric properties to fine-tune the extent of PeT and consequently, fluorescence lifetimes. Zhang *et al.* synthesized a library of polarity- and viscosity-insensitive BODIPY fluorophores with various substituted aromatic groups at the 8-position (Fig. 2).^37^ The authors demonstrated that decreasing the electron-withdrawing or electron-donating ability of the *para*-substituents (*e.g.*, 1a (cyano): 1.71 ns *vs.*1b (fluoro): 3.78 ns; 1c (hydroxyl): 3.36 ns *vs.*1d (methyl): 3.74 ns; all values measured in ethanol), and increasing the steric hindrance at the *ortho*-position (1b (*para*): 3.78 ns *vs.*1e (*ortho*): 6.14 ns; all values measured in ethanol) led to gradually longer fluorescence lifetimes.

Beyond the substituents at the *meso*-position, chemical modifications at the α, β and γ positions of the BODIPY core can also effectively modulate its fluorescence lifetime. For the same *meso*-substituents, α,γ-methyl-substituted green BODIPY 1f exhibited near complete fluorescence quenching with no viscosity or polarity sensitivity due to strong PeT effect (Fig. 2),^37^ but this was not observed for β-phenyl-substituted red BODIPY 2, where installation of the same *para*-nitro group could increase its dynamic range (expressed here as a lifetime ratio at high and low viscosities) in castor oil/water mixtures by more than three-fold from 1.4 to 4.6.^38^ Other red-emitting BODIPY derivatives 3 have also been synthesized by the extension of π-conjugation with aryl groups or heterocycles at position 5.^37^ In this case, PeT plays a minimal role and lifetime modulation is primarily governed by ICT mechanisms. Due to the differences in excited-state energy transfer mechanisms, electron-deficient groups (3a (cyano): 5.19 ns in ethanol) at the 5-position exhibited longer lifetimes than electron-rich substituents (3b (dimethylamino): 3.79 ns in ethanol), unlike those observed for the *meso*-position. In another strategy, methyl groups can be added to positions 1 and 7 to restrict the free rotation of *meso*-substituents and extend fluorescence lifetimes,^39^ although this chemical modification can reduce viscosity sensitivity.^40^ Altogether, these chemical strategies illustrate how electronic and steric modulation at different positions of the BODIPY core can be used to modulate the fluorescence lifetimes of its derivatives.

Like BODIPYs, rhodamines are well-established fluorophores widely used in bioimaging due to their high fluorescence quantum yields, brightness, photostability and excellent biocompatibility.^41–43^ Their modular structure also allows for structural modifications to modulate fluorescence lifetimes *via* similar energy transfer processes. Urano *et al.* synthesized pH-sensitive rhodamine 4 by introducing electron-donating anilines to the pendant phenyl ring,^44^ which enabled lifetime control by modulation of PeT efficiency (Fig. 2). *para*-Substitution of the N-substituent (4a: 1.40–2.10 ns) yielded rhodamines with pH-sensitive lifetimes (long *τ* at pH 2.0, short *τ* at pH 9.0) compared to *meta*- (4b: 2.13–2.20 ns) or *ortho*- (4c: 2.07–2.18 ns) substituted analogues. Furthermore, the addition of a methyl group to the aniline N-substituent (4d: 0.24 to 2.00 ns) further increased the lifetime dynamic range from its non-methylated derivative 4a, improving the pH responsiveness of the probes.

The auxochromes of rhodamines, which are typically electron-donating dialkylamino groups, can also be fine-tuned to modulate fluorescence lifetimes. The introduction of geometrically constrained azacyclic auxochromes (*e.g.*, azetidine 5a: 4.69 ns in phosphate buffered saline (PBS)) is a well-studied method to inhibit TICT,^45,46^ which increases quantum yields and fluorescence lifetimes. This strategy is not as effective for red to NIR carborhodamines and silicon-rhodamines^47^ but can be mitigated *via* the introduction of electron-withdrawing groups to the amino auxochrome. Guo *et al.* demonstrated that the inductive effect strength of electron-withdrawing groups such as sulfone groups (5b: 5.89 ns in PBS) inhibits TICT to improve quantum yields and fluorescence lifetimes.^48^ On the other hand, increasing TICT propensity may improve environmental sensitivity, which can be achieved by replacing one of the *N*,*N*-dialkyl auxochrome groups with pyrrole derivatives.^49^ Using this strategy, Fang *et al.* synthesized dye 6a, which exhibited an increased dynamic range (0.74–3.54 ns) and enhanced micropolarity sensitivity in DCM/methanol mixtures compared to the commercially available rhodamine B (6b, 2.34–4.34 ns).^50^ However, this was accompanied by an evident decrease in overall lifetimes, illustrating the trade-off between lifetime extension and environmental sensitivity. Precise auxochrome engineering is therefore an effective strategy to balance lifetime, brightness, and responsiveness in developing rhodamine probes for lifetime imaging.

Cyanine dyes, characterised by their linear polymethine chains flanked by two heteroaromatic nitrogen atoms, are widely used in bioimaging applications due to their spectral tunability and emission in the visible and NIR regions.^51^ Notably, cyanines are one of the few fluorophores that extend into the short-wave infrared region, enhancing tissue penetration for *in vivo* imaging.^52^ To tune lifetimes of such dyes, Štacko *et al.* introduced deuteration as a strategy to extend the fluorescence lifetimes of Cy7 dyes.^53^ Replacement of hydrogen atoms with the heavier deuterium atom results in stiffer and less energetic C–D stretching, altering the vibrational mode of the fluorophore. As less energy is lost by non-radiative vibrations, deuterated 7a exhibits an increased lifetime (547 ps) compared non-deuterated 7b (481 ps) (Fig. 2). Like 7, conventional cyanines exhibit short fluorescence lifetimes around 1 ns or less due to their flexible molecular structure, which facilitates high rates of non-radiative decay *via* excited state *trans*-to-*cis* photoisomerisation.^54^ To suppress excited-state relaxation pathways, Schnermann *et al.* reported a conformationally-restraining ring system to produce di-sulfonated cyanine 8. The cyanine 8 possesses a higher quantum yield and a longer lifetime of 1.89 ns compared to its unrestrained counterpart Cy5 (1.09 ns in PBS),^55^ highlighting the effectiveness of structural rigidification in tuning fluorescence lifetimes.

Nitrobenzodiazoles (NBDs) are attractive fluorophores for biological imaging because of their low molecular weight and permeability.^56,57^ Their intrinsic ICT character stems from their donor–π–acceptor (D–π–A) structure, which makes their fluorescence emission and lifetimes highly sensitive to the local environments (*e.g.*, polarity, viscosity). Lifetime modulation of NBD fluorophores by synthetic methods focuses on controlling the strength of ICT, specifically by altering the donor (typically amino) and acceptor (typically nitro) groups at positions 4 and 7 of the core. For example, Guo *et al.* synthesized 9 by installing the strong electron-donating *N*,*N*-dimethyl group and electron-withdrawing sulfonamide group to maximise the extent of ICT and substantially extend the dynamic range of lifetimes above 15 ns (Fig. 2, 0.80 ns to 16.62 ns in 1,4-dioxane/water mixtures).^58^ The modification of the bridging heteroatoms in NBD fluorophores from sulfur (10a: 9.62 ns in 1,4-dioxane) to selenium (10b: 5.65 ns in 1,4-dioxane) can also alter conjugation and spin–orbit coupling to yield distinct fluorescence lifetimes.^59^

Other families of fluorophores such as napthalimides^60^ and triangulenium dyes^61^ have also been explored for lifetime-based imaging. Across these structures, the introduction of substituents at specific sites can result in effective modulation of fluorescence lifetimes. For instance, in quinoliziniums, the introduction of electron-donating substituents such as methoxy (11b: 2.6 ns in DMSO) and hydroxyl groups (11c: 0.3 ns in DMSO) shortened the fluorescence lifetimes of pyranoquinolizinium 11a (5.2 ns in DMSO) when introduced at the R position (Fig. 2), likely due to the destabilisation of its excited state.^62^ In contrast, for Nile Red (9-diethylaminobenzo[*a*]phenoxazin-5-ones) analogues, the chemical modification of positions 1, 2, 3, 4, 10 and 11 led to minimal lifetime changes in non-polar environments (*e.g.*, toluene, chloroform) but exhibited large lifetime changes in polar environments (*e.g.*, methanol) as evidenced by the comparison between Nile Red 12a (>4.0 ns in toluene or chloroform, 2.8 ns in methanol) and its fluorinated derivative 12b (>3.8 ns in toluene or chloroform, 0.5 ns in methanol).^63^ These distinct changes in lifetime enable the Nile Red derivatives to be used as lifetime-based polarity sensors of lipid domains. Triangulenium dyes exhibit long fluorescence lifetimes (>10 ns) and emission in the red channel due to their extended conjugation over a planar structure, making them interesting fluorophores for FLIM. Within this family, the inclusion of tertiary amine groups as side chains on the dye scaffold, such as aminoethyl morpholine, has been found to alter the fluorophore's response to different nucleic acid topologies.^64^ This environmental sensitivity is driven by PeT quenching, which is alleviated to varying extents in different spatial arrangements of nucleobases.

### Modification of fluorescence lifetime by external stimuli

Due to its dependency on *k*_nr_, fluorescence lifetimes are typically sensitive to local microenvironments, making it a powerful readout for dynamic biological processes. To take advantage of this property, stimuli-responsive fluorophores have been designed to show lifetime changes in response to external factors such as biomolecular, biophysical or biochemical interactions. These approaches form the foundation of dynamic lifetime imaging, enabling selective and spatially resolved measurements of biological activity.

**Fig. 3:**
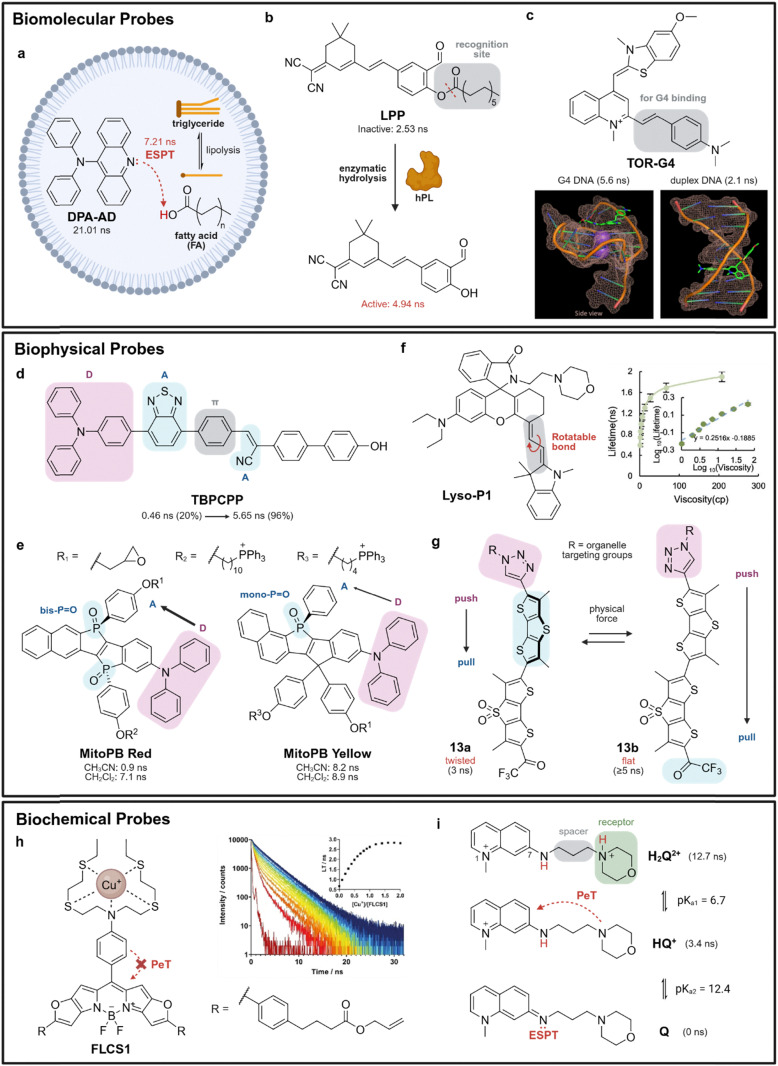
Fluorescence lifetime modulation by biomolecular, biophysical and biochemical stimulation. (a) Schematic of DPA-AD undergoing excited-state proton transfer (ESPT) with fatty acids (FAs) derived from the lipolysis of triglycerides in lipid droplets. (b) Mechanism depicting the release of active LPP*via* enzymatic hydrolysis at the recognition site (red dotted line) by human pancreatic lipase (hPL). (c) Structure of TOR-G4 featuring an extended π-conjugation (grey) for improved G4 binding. Molecular modeling of TOR-G4 bound to the G-quadruplex c-MYC or duplex DNA structures demonstrates variations in the probe's binding conformation and corresponding lifetime changes. Reproduced with permission from ref. 72. Copyright 2024, American Chemical Society. (d) D–A–π–A structure of TBPCPP confers polarity-sensitive lifetimes in 1,4-dioxane/methanol mixtures (20% to 96% 1,4-dioxane). (e) Structures of MitoPB Red (bis-P=O) and MitoPB Yellow (mono-P=O) with donor (D) and acceptor (A) groups highlighted in purple and blue respectively. The bis-P=O structure increases the dipole moment from D to A compared to mono-P=O, improving the sensitivity of MitoPB Red to polar solvents (CH_3_CN). (f) Structure of Lyso-P1 featuring a rotatable π-bridge (grey) between the xanthene core and heterocyclic substituent for viscosity-sensitive lifetimes (graph). Reproduced with permission from ref. 81. Copyright 2024, American Chemical Society. (g) Mechanism behind lifetime modulation of HydroFlipper 13. Physical force causes conformational flattening of one of the two dithienothiophene fluorophores in 13a (blue), which extends the push–pull system in 13b from the triazole donor (purple) to the trifluoroketone acceptor (blue). (h) Structure of FLCS1 featuring an NS_4_ copper(i) receptor. Binding of Cu^+^ ions to FLCS1 decreases PeT efficiency, leading to an increase in lifetime (graph). Reproduced with permission from ref. 85. Copyright 2021, Wiley-VCH. (i) Structures of H_2_Q^2+^, HQ^+^, and Q feature an alkyl spacer and a morpholine pH receptor. Initial proton dissociation of H_2_Q^2+^ to HQ^+^ shortens lifetimes due to PeT by the morpholine moiety. Subsequent proton dissociation of HQ^+^ to Q shortens lifetimes further due to ESPT at the 7-amino moiety.

Biomolecular probes display changes in lifetimes after they react or bind to molecular targets. A key feature in the design of biomolecular probes is the incorporation of target-responsive elements that can modulate excited-state processes upon interaction with specific biomolecules. For example, Zhang *et al.* synthesized DPA-AD, an acridine-based probe able to differentiate fatty acids (FAs) from triglycerides in lipid droplets (LDs) (Fig. 3a).^65^ Its design leverages on excited-state proton transfer (ESPT), which occurs between the carboxylic acid moiety of FAs and the N atoms of the acridine group. This interaction enhances non-radiative decay and shortens the fluorescence lifetime of acridine substantially from 21.01 ns, when only tricaprylin (a typical LD triglyceride) is present, to 7.21 ns, upon the recognition of FAs such as oleic acid. Furthermore, enzyme-activatable lifetime probes have also been developed to detect the activities of enzymes such as human pancreatic lipase (hPL)^66^ and non-receptor tyrosine protein kinases (*e.g.*, Abl and Src families).^67,68^ For probes like LPP,^66^ enzymatic hydrolysis by hPL (Fig. 3b) converts the fluorophore from an inactive state (2.53 ns) to an active state (4.94 ns) to effectively sense enzyme activity in cells.

In addition to reaction-based lifetime modulation, the non-covalent binding of a probe to its target biomolecule can alter its conformation and the non-radiative pathways in the excited states.^69^ This is a common strategy employed to study DNA and histone packing, such as imaging of G-quadruplex nucleic acids (*e.g.*, G4 RNA, G4 DNA) in live cells using fluorescence lifetime imaging microscopy (FLIM).^70,71^ Kuimova *et al.* synthesized a thiazole orange (TO) derivative TOR-G4 with a benzyl-styryl unit to extend the π-conjugation of the TO core for binding to G-quartets.^72^ Molecular modelling revealed that TOR-G4 binds to G4 DNA *via* end-stacking, but intercalates into duplex DNA, resulting in significantly different lifetimes for each binding mode (5.6 ns for G4 DNA c-MYC *vs.* 2.1 ns for duplex DNA) (Fig. 3c). Remarkably, this strategy is not limited to targeting nucleic acids, but also applicable to protein aggregates. Aggregation restricts the intramolecular motion of rhodamine fluorophores like NRhFluors,^73^ which reduces non-radiative decay and increases the lifetimes of aggregation-induced emissive probes (*e.g.*, 3.05 to 3.51 ns during heat-induced protein aggregation) to monitor protein conformational changes.

Biophysical probes can be designed to exhibit lifetime changes when they are affected by external factors, like polarity and viscosity. Their ability to distinguish between local microenvironments arises from environment-sensitive excited-state mechanisms, such as ICT, TICT, non-radiative torsional relaxation and hydrogen bond-induced quenching.^74^ In micropolarity sensing probes, push–pull structures with D–π–A or similar systems are commonly employed as their intrinsic ICT mechanisms confer enhanced sensitivity to variations in the local dielectric constant or hydrophobicity. Aside from conventional D–π–A NBDs,^75^ Wang *et al.* developed TBPCPP which consisted of triphenylamine (D), benzene (π), 2,1,3-benzothiadiazole (A) and acrylonitrile (A) to establish a D–A–π–A structure (Fig. 3d).^76^ In 1,4-dioxane/methanol mixtures, TBPCPP displayed polarity-dependent fluorescence lifetimes from 0.46 to 5.65 ns as the 1,4-dioxane fraction increased from 20% to 96%. For superior sensitivity against polarity, the excited-state dipole moment of the fluorophore must be sufficiently large to facilitate stronger interactions with solvent molecules. This can be achieved by tuning the electron donating or accepting strength of the donor and acceptor groups respectively. For example, to develop the D–π–A fluorophore MitoPB Red, Yamaguchi *et al.* introduced two single arene-fused phosphole *P*-oxide (bis-P=O) as electron acceptors (A) (Fig. 3e).^77^ This significantly increased its sensitivity to polarity (0.9 ns in polar acetonitrile, 7.1 ns in non-polar DCM) compared to mono-P=O counterparts (*e.g.*, MitoPB Yellow, 8.2 ns in polar acetonitrile, 8.9 ns in non-polar DCM), enabling a higher extent of ICT to amplify lifetime response to micropolarity.

To leverage non-radiative processes for microviscosity sensing, molecular rotors with flexible or twisted conformations can be designed by incorporating a flexible bond or rotatable group that allows for TICT or rotational relaxation to influence lifetime changes under different viscosity conditions. Several molecular rotors have been designed using BODIPYs and rhodamines.^78–80^ Shen *et al.* designed a rhodamine-based lysosome-targeting probe, Lyso-P1, that exhibited microviscosity-dependent lifetimes.^81^ The main feature of Lyso-P1 is a π-bridge that lies between the xanthene core and its π-excessive heterocyclic substituent (Fig. 3f). It acts as a rotatable bond that allows for rotational relaxation in non-viscous environments, shortening lifetimes from 1.91 to 0.59 ns as viscosity decreases from 208 cP to 1 cP. Alternatively, planarizable push–pull systems, such as the HydroFlippers 13 developed by Matile *et al.*, can detect variations in mechanical tension and hydration of membranes of interest.^82,83^ These probes are designed to undergo conformational flattening in ordered lipid environments (Fig. 3g), enhancing π-conjugation to extend lifetime (13a twisted: 3 ns, 13b flat: ≥5 ns) *via* changes in ICT. Other ICT-dependent lifetime probes for membrane hydration imaging have also been developed using conjugated oligoelectrolytes to leverage its D–π–A structure.^84^

Beyond physical stimuli, biochemical probes can respond to chemical parameters such as pH and ion concentration. Such probes typically consist of protonatable groups or ion-binding moieties that shift the balance of excited-state processes, such as PeT and ESPT when interacting with protons or metal ions. For example, Vilar *et al.* reported FLCS1 as a BODIPY fluorophore to sense Cu^+^ ions in solution.^85^FLCS1 featured a *para*-substituted electron-rich NS_4_ copper(i) receptor at the 8-position of the BODIPY core. This fluorophore was quenched to about 0.5 ns in the absence of Cu^+^ due to PeT, whereas binding to Cu^+^ ions reduced the electron-donating ability of the NS_4_ chelating fragment and minimised PeT to increase the fluorescence lifetime to about 2.5 ns (Fig. 3h). Interestingly, for pH sensing, there are limited reports on pH-dependent FLIM sensors with a good dynamic range and linear response to pH. Nevertheless, Jager *et al.* developed H_2_Q^2+^ based on the 1-methyl-7-amino-quinolinium fluorophore to detect high pH values.^86^H_2_Q^2+^ was designed to include an additional ionizable group in the form of a spacer-receptor moiety attached to the 7-position (Fig. 3i) to fine-tune both the probe's p*K*_a_ and lifetime values. While the initial deprotonation to HQ^+^ at p*K*_a1_ = 6.7 causes a decrease in lifetime (12.7 to 3.4 ns) due to PeT quenching by the morpholine receptor, ESPT at the 7-position amino group serves as the main mechanism behind the observed shortened lifetime (3.4 ns to non-detectable lifetimes) at the next deprotonation to Q at p*K*_a2_ = 12.4. While H_2_Q^2+^ may face limited applications for FLIM imaging due to its blue emission profile, other pH-sensitive FLIM probes can also be functionalised with specific targeting motifs such as CarboTag, a small molecule that binds to diols in the plant cell wall with high affinity, to expand the scope of such fluorophores in FLIM.^87^

Collectively, the synthetic approaches employed across different fluorophores, namely the introduction of substituents and conformational rigidification, as well as the introduction of design elements responsive to biomolecular, biophysical and biochemical stimuli serve as powerful methods to tailor fluorescence lifetimes for diverse imaging applications. Moving forward, computational approaches such as time-dependent density functional theory (TD-DFT) are increasingly being used to describe electronic distributions and charge-transfer characteristics relevant to lifetime modulation.^88^ It is important to note that current TD-DFT approaches face limitations in accurate *k*_nr_ determination, especially when solvent effects are present. On the other hand, such computational insights can complement experimental data, particularly for evaluating trends across related fluorophore scaffolds, to guide and accelerate the rational design of lifetime probes for imaging studies.

## Novel chemical probes for FLIM

FLIM is an optical imaging modality that generates spatial maps of fluorescence lifetimes. Unlike intensity-based imaging, fluorescence lifetimes are minimally affected by fluorophore concentration and laser excitation power, which enables robust quantitative and ratiometric imaging.^89^ Fluorescence lifetimes are also sensitive to local microenvironments, providing additional information of dynamic cellular processes. The development of chemical probes for FLIM has enabled the real-time visualisation of previously inaccessible cellular mechanisms, such as biomolecular condensate regulation and membrane alterations during ferroptosis. Furthermore, FLIM probes can track lifetime variations between healthy and diseased states, paving the way for new diagnostics that detect early pathological changes and monitor treatment responses. In this section, we discuss the application of novel chemical FLIM probes in mechanistic studies and disease diagnosis.

## Chemical probes for imaging of cellular dynamics and biomolecular interactions

Detailed knowledge of cellular dynamics and biomolecular interactions is instrumental for the successful design of therapeutics and diagnostics.^90^ Due to their environmental sensitivity, FLIM chemical probes are an attractive approach to image organelles, biomolecules and signaling pathways as well as to provide insights into cellular microenvironments *via* the monitoring of fluorescence lifetimes.^91–93^ This advantage positions FLIM as a powerful technique for the elucidation of cellular mechanisms and processes beyond the capabilities of conventional fluorescence imaging.

**Fig. 4:**
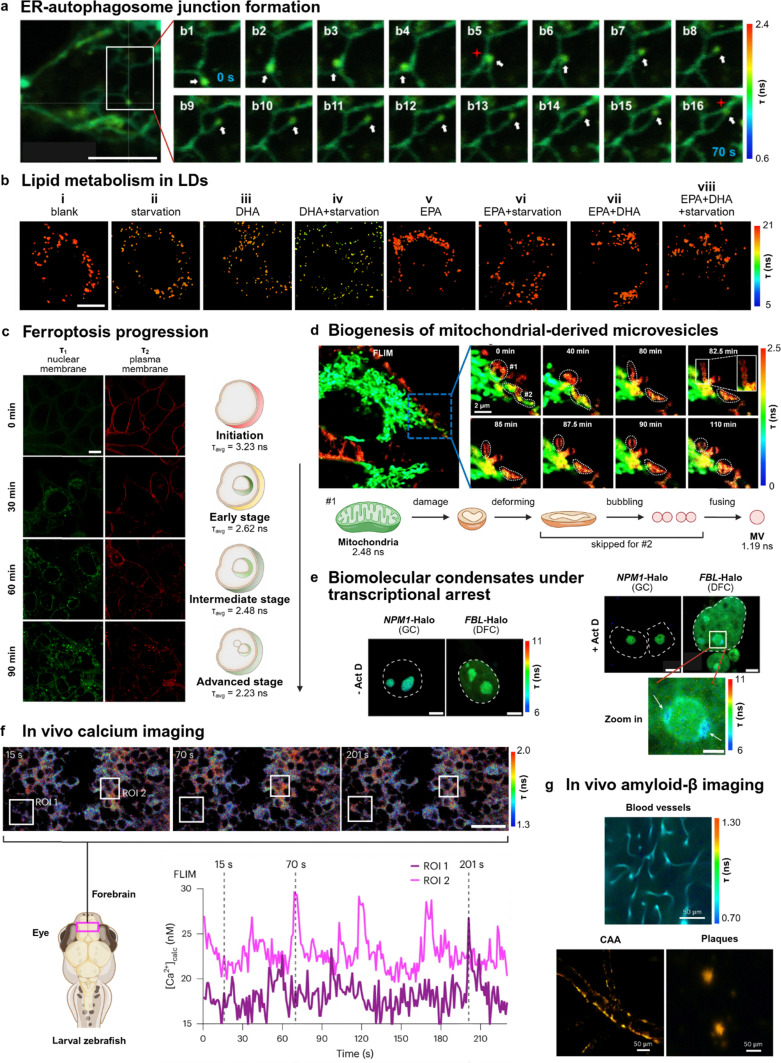
FLIM imaging of cellular dynamics under healthy and diseased states. (a) FLIM imaging of dynamic ER tubules coupled to motile autophagosomes in 3T3 cells stained with 5 µM CF2. The white frame marks the site of the observed coupling while the red stars label the three-way junction formed between the ER tubule and autophagosome. The growing tip of a newly formed autophagosome-associated ER tubule (white arrows) was monitored over 70 s. Scale bar: 5 µm. Reproduced with permission from ref. 95. Copyright 2022, American Chemical Society. (b) FLIM imaging of omega-3 FAs effect on triglyceride lipolysis. HepG2 cells were treated with: (i) DPA-AD for 20 min; (ii) DPA-AD for 20 min, then serum-free media for 2 h; (iii) DHA for 2 h, then (i); (iv) DHA for 2 h, then (ii); (v) EPA for 2 h, then (i); (vi) EPA for 2 h, then (ii); (vii) DHA and EPA for 2 h, then (i); (viii) DHA and EPA for 2 h, then (ii). All concentrations of DPA-AD and FAs (DHA/EPA) are 2 µM or 10 µM respectively unless otherwise stated. A decrease in lifetime corresponds to an increase in FA concentration and lipolysis activity in LDs. Scale bar: 10 µm. Reproduced with permission from ref. 65. Copyright 2024, Proceedings of the National Academy of Sciences. (c) FLIM images of double exponential fitting components *τ*_1_ and *τ*_2_ of ferroptotic cells. 4T1 cells were treated with 10 µM of erastin under various durations followed by 10 µM of DF1 to monitor changes in membrane lifetimes at different stages of ferroptosis illustrated by the adjacent schematic. Scale bar: 10 µm. Reproduced with permission from ref. 98. Copyright 2024, National Academy of Sciences. (d) FLIM images and schematic of the biogenesis of mitochondria-derived MVs by IC-C4. SH-SY5Y cells were incubated with 0.2 µM of IC-C4, followed by treatment with 10 µM of CCCP. Scale bar: 2 µm. Reproduced with permission from ref. 101. Copyright 2025, American Chemical Society. (e) FLIM images of HEK293T cells expressing NPM1-Halo or FBL-Halo labeled by S-SBD-Halo in the absence and presence of 0.2 µM Act D. Scale bars: 2 µm (zoom in), 5 µm (others). Reproduced with permission from ref. 102. Copyright 2024, Springer Nature. (f) FLIM of WHaloCaMP in live zebrafish larvae showing spontaneous neuronal activity in the forebrain. Ca^2+^ concentrations calculated from a FLIM calibration curve over time for two neurons (ROI 1 and 2) in the forebrain of zebrafish larvae are shown in the graph. Scale bar: 20 µm. Reproduced with permission from ref. 104. Copyright 2024, Springer Nature. (g) FLIM images of ZW800-1C showing blood vessels, CAA, and amyloid plaque labelling 2 h post-injection. Scale bar: 50 µm. Reproduced with permission from ref. 108. Copyright 2023, Springer Nature.

FLIM probes have been employed to study the interactions between the endomembrane system and associated organelles. These include the endoplasmic reticulum (ER) and LDs, whose dysregulation are linked to the onset of pathological states.^94^ Lin *et al.* described CF2 as a dual fluorescence lifetime coumarin-based probe that responded to membrane tension, enabling one the first imaging studies of ER-autophagosome dynamics.^95^CF2 functions as a molecular rotor and exhibits shorter lifetimes (1.65 ns) in the ER and longer lifetimes (1.90 ns) in autophagosomes due to differences in membrane tension. CF2 (Fig. 4a) revealed coupling between ER tubules and autophagosomes (white arrows) through three-way junction formation (red stars in b5 and b16) over 70 s and was also used to track ER autophagy during cisplatin-induced apoptosis, uncovering a possible relationship between intensive autophagy and apoptotic pathways. Other forms of ER autophagy have also been monitored using FLIM. Notably, probes such as RHC revealed that Tat-beclin 1 (TB)-induced ER autophagy was partially inhibited by triglycerides,^96^ highlighting the functional link between lipid metabolism and ER regeneration. Building on this connection, LD dynamics and triglyceride metabolism were explored using DPA-AD, a FLIM-compatible activity-based probe that exhibits short and long lifetimes when levels of FAs in LDs are high and low respectively.^65^ Aside from visualizing LD fusion, fission and lipolysis under different nutrient conditions, DPA-AD revealed the counteracting effects of the omega-3 FAs docosahexaenoic acid (DHA) and eicosapentaenoic acid (EPA) commonly found in fish oil supplements.^65^ Such supplements have traditionally been thought to increase the rate of triglyceride breakdown to fatty acids, which is supported by the DHA's ability to increase the level of FAs in LDs under both normal and starved states (Fig. 4b, i–iv). However, the addition of EPA or coaddition of DHA and EPA to HepG2 cells did not affect the FA level even under normal or starved conditions (Fig. 4b, v–viii). These results suggest that EPA could play a role in buffering intracellular lipolysis to maintain a stable pool of FAs, providing new insights into lipid metabolism and the prevention of cardiovascular diseases *via* nutritional means.

Aside from regulatory organelle mechanisms, the dynamics of cell death mechanisms such as ferroptosis have been studied using FLIM chemical probes.^97^ Lin *et al.* reported the plasma membrane and nuclear membrane-binding TICT probe DF1 to investigate cells undergoing erastin-induced ferroptosis.^98^ By monitoring real-time changes in fluorescence lifetimes (*τ*_1_ and *τ*_2_) and membrane morphology, they proposed a three-stage progression for ferroptosis (Fig. 4c). Specifically, DF1, originally poorly permeable to the plasma membrane (0 min, initiation), was able to enter the 4T1 cells (early stage) and report an increase in the *τ*_1_ component corresponding to the nuclear membrane (intermediate stage). This was followed by budding of the nuclear membrane (advanced stage), with an overall decrease in *τ*_avg_ from 3.23 ns to 2.23 ns over 90 min, suggesting an increase in membrane tension during ferroptosis. Aside from membrane dynamics, FLIM images using another probe CQPP revealed that cells undergoing ferroptosis exhibited different changes in lifetimes in LDs and the nucleus compared to apoptotic cells, which suggest differing mechanistic pathways in these cell death processes.^99^ These FLIM probes can enhance our understanding of not only complex cell death pathways but also organelle dynamics in perturbed cellular contexts such as photodynamic therapy,^100^ which highlights their potential therapeutic target validation in cell biology studies.

In recent years, microvesicles (MVs) and biomolecular condensates have emerged as potential therapeutic targets due to their role in regulating cell function. Unfortunately, these compartments are challenging to image because of the lack of biomarkers and their transient nature. To overcome this, Zhang *et al.* developed IC-C1 as a cationic FLIM probe to image MV dynamics, including fusion, fission, plasma membrane shedding, and pseudopodium-dependent motility. Using the same fluorophore core, the authors also synthesized IC-C4 to label both MVs (2.48 ns) and mitochondria (1.19 ns) with different fluorescence lifetimes and provide information on the biogenesis of mitochondria-derived MVs during carbonyl cyanide 3-chlorophenylhydrazone (CCCP)-induced mitochondria damage (Fig. 4d).^101^ Notably, the authors found that these mitochondria-derived MVs were not secreted but instead directly merged with lysosomes, revealing a potential pathway for eliminating dysfunctional mitochondria in living cells. In another study, Zhang *et al.* demonstrated that the structural organisation of nuclear biomolecular condensates was dependent on local micropolarity.^102^ Using an environmentally sensitive NBD-based probe S-SBD-Halo, they associated micropolarity changes to the translocation of the inner dense fibrillar component (DFC) layer outside of the granular component (GC) layer to form nucleolar caps (white arrows) during actinomycin D (Act D)-induced transcriptional arrest in the nucleolus (Fig. 4e). This biophysical process is also influenced by ion-species effects on the condensate microenvironment,^103^ providing new mechanistic insights into the structural regulation and environmental response of biomolecular condensates.

Cellular messengers like Ca^2+^ ions and cyclic adenosine monophosphate (cAMP) form the basis of effective cellular communication and their visualisation can provide useful insights into many biological processes. To address the bioavailability challenges associated with *in vivo* Ca^2+^ imaging of the brain, Schreiter *et al.* designed a WHaloCaMP, a chemigenetic indicator for Ca^2+^ ions that combined a rhodamine fluorophore with PeT-based quenching to selectively visualise Ca^2+^ in the forebrain of larval zebrafish.^104^ Using FLIM, they demonstrated real-time quantitative Ca^2+^ imaging in populations of forebrain neurons, enabling single-cell resolution tracking of absolute intracellular Ca^2+^ concentrations (Fig. 4f). Lavis *et al.* also designed a semisynthetic construct combining the self-labelling HaloTag and SNAP-tag systems with rhodamine derivatives to perform FRET-based lifetime imaging of cAMP.^105^ Beyond the main signaling players, additional FLIM-compatible chemical probes have been reported for quantitative sensing of other signaling messengers such as Al^3+^ ions and free radicals.^106,107^ Taken together, FLIM-based probes allow monitoring of cellular dynamics and biomolecular interactions with high spatiotemporal resolution. This feature has not only enabled quantitative monitoring of biomarkers associated with cell function but also provided avenues for potential therapeutic target validation in cell biology.

### Microenvironment-sensitive chemical probes for disease diagnostics

The development of accurate diagnostic tools is indispensable for the effective treatment of diseases. Such tools require comprehensive understanding of both healthy and pathological states. Current image-based diagnostics include computed X-ray tomography, magnetic resonance imaging and ultrasonic imaging;^16^ however, the unique advantages and improvements to multiplexing capabilities^109–111^ make FLIM a promising modality for acquiring complex molecular signatures during the progression of neurodegenerative disorders, metabolic diseases and cancer.^31^

A key neurodegenerative disorder is Alzheimer's disease (AD), which is characterised by the aberrant assembly of amyloid-β (Aβ) peptides that leads to insoluble Aβ oligomer formation and neurotoxic Aβ plaque accumulation in the brain. Unfortunately, AD lacks early diagnostic tools,^112,113^ which hampers timely intervention for effective management of the disease. To this end, efforts have been made to develop probes for Aβ fibrils showing longer fluorescence lifetimes than Aβ monomers and allowing for lifetime-based identification of Aβ aggregates in cells.^114,115^ Choi *et al.* developed the near-infrared zwitterionic heptamethine fluorophore ZW800-1C, which binds to Aβ aggregates for *in vivo* imaging in mice.^116^ Intravenous injection of ZW800-1C followed by *in vivo* FLIM imaging was employed to detect cerebral amyloid angiopathy (CAA, 1.27 ns), a form of Aβ deposit on the blood vessels of the brain, as well as Aβ plaques (1.24 ns), which displayed longer lifetimes than unbound ZW800-1C in blood vessels (0.84 ns) (Fig. 4g).^108^ This probe offers a promising non-invasive imaging modality for studies of AD in mouse models and with further potential for multiplexed fluorescence lifetime tomographic imaging to simultaneously monitor several biomarkers of disease.^117^

For metabolic disorders like type 2 diabetes, tissue inflammation is a common marker of both acute and chronic onset of the disease, leading to complications such as organ damage.^118^ As inflammation is associated with an increase in tissue and intracellular viscosity,^119^ Lin *et al.* designed the microviscosity-sensitive xanthene-based probe YF-V to detect changes in viscosity in organs affected by diabetes.^120^YF-V exhibited longer fluorescence lifetimes within HL-7702 cells treated with high glucose, indicative of high intracellular viscosity. Lifetime imaging of harvested spleen and blood vessel tissues from diabetic mice also suggested that YF-V could be used to monitor hyperglycemia-induced viscosity changes in these tissues over a period of seven days (spleen: 0.93 ns to 1.35 ns, blood vessel: 1.10 ns to 1.68 ns). Furthermore, organs such as the lung, heart, brain, liver and kidney developed abnormal viscosity changes in a long-term diabetic state (15 days), providing insights into diabetic complications.

Cancer imaging using FLIM typically employ the use of endogenous fluorophores (*e.g.*, nicotinamide adenine dinucleotide (NAD(P)H)),^121,122^ due to their ability to reveal changes in intracellular metabolism.^123^ Although reports of chemical probes for cancer diagnosis using FLIM are limited, Shirmanova *et al.* demonstrated the use of a plasma membrane-targeting BODIPY fluorophore to visualise differences in viscosity between cisplatin-resistant and non-resistant cancer cells.^124^ Such probes offer valuable potential to understand the mechanisms behind drug treatment in cancer cells and aid design of tailored anti-cancer treatments.

Looking forward, lifetime imaging of inflammation, which is a hallmark of numerous pathologies including neurodegeneration, metabolic diseases and cancer, represents a particular promising frontier. While FLIM applications for imaging inflammation are still emerging,^125^ complementing the use of endogenous fluorophores with exogenous probes that detect additional biomarkers could improve early-disease diagnosis, treatment monitoring and mechanistic studies. By leveraging the advantages of FLIM and chemical probes, namely concentration-independent readouts, micro-environmental sensitivity and multiplexing capability, deeper insights into various disease processes can be gained for transformation into more effective therapeutic approaches.

### Approaches for the acquisition and analysis of FLIM data

The ability of FLIM to provide quantitative insights into biomolecular interactions, cellular microenvironments and dynamic processes is not only dependent on the performance of the chemical probes but also on the methodologies for acquisition and data analysis. FLIM was initially limited by long acquisition times, which hindered its application in high-throughput studies or in dynamic live-cell imaging.^26–28^ Recent advances in hardware, photon detection strategies and computational methods including artificial intelligence (AI), have significantly enhanced FLIM performance by enabling real-time, high-content and multiplexed imaging.^126^ These improvements fall under two major domains: (1) data acquisition techniques, including time-domain and frequency-domain methods and (2) computational data analysis strategies, including machine-learning assisted lifetime reconstruction.

### Innovations in FLIM acquisition techniques

**Fig. 5:**
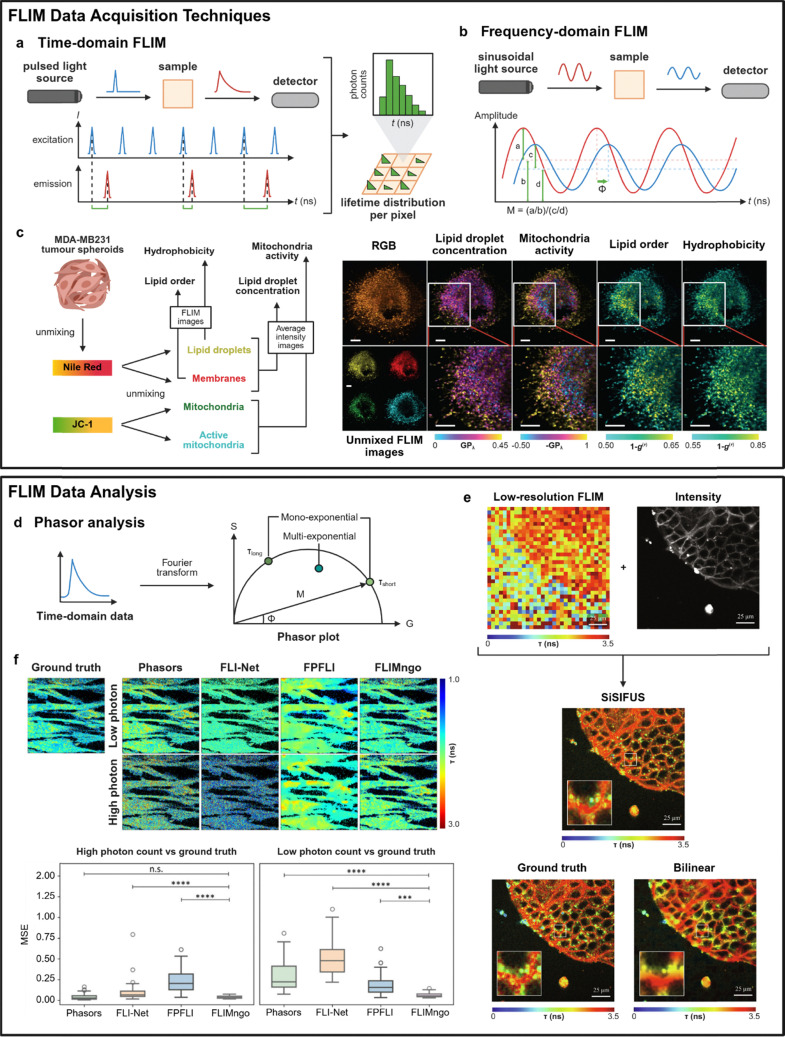
Current and emerging FLIM data acquisition and data analysis techniques. (a) Simplified schematic of the working principles of time-domain FLIM. (b) Simplified schematic of the working principles of frequency-domain FLIM. (c) Schematic representation of Phasor S-FLIM enabling the imaging (FLIM/intensity) of key metabolic parameters. MDA-MB231 tumor spheroids are labeled with Nile Red and JC-1 as shown in the RGB image. Using the calibrated spectral signature of the dyes, four FLIM images are unmixed. The unmixed images are associated with lipid droplets (top left), internal membranes (top right), mitochondria (bottom left) and active mitochondria (bottom right). Average intensity images are combined to obtain GP_λ_ maps corresponding to lipid droplet concentration and mitochondria activity. FLIM images of the Nile Red spectral emission are used to map lipid order of the internal membranes and hydrophobicity of the lipid droplets. For both FLIM and GP_λ_ images, a larger value indicates higher lipid droplet concentration, mitochondrial activity, lipid order, or hydrophobicity. Scale bars: 50 µm. Reproduced with permission from ref. 137. Copyright 2021, Springer Nature. (d) Schematic showing key characteristics of a phasor plot during phasor analysis. Frequency-domain data (*M*, *Φ*) and time-domain data (processed by Fourier transform) can be used for phasor analysis. On the phasor plot, data points on the curve indicate mono-exponential decays, while points within the semicircle are multi-exponential in nature. Points on the curve closer to the left have a longer lifetime while those closer to the right have a shorter lifetime. (e) Upsampling of MDCK cells expressing flipper-TR dye. Data from a low-resolution FLIM image and its corresponding high-resolution intensity image were fused using SiSIFUS to reconstruct a high-resolution FLIM image. The ground truth high-resolution FLIM image was obtained using least-squares deconvolution. Scale bars: 25 µm. Reproduced with permission from ref. 150. Copyright 2024, American Association for the Advancement of Science. (f) Fluorescence lifetime prediction by various data analysis techniques across photon count conditions. Fluorescence lifetime maps of the ground truth (identical for low and high photon counts) alongside the maps predicted by phasor plot analysis, FLI-Net, FPFLI, and FLIMngo. Box-and-whisker plots of the MSE scores for predicted fluorescence lifetime maps from high and low photon counts compared to ground truth data. Statistical significance was calculated using a Kruskal–Wallis test followed by Dunn's multiple comparisons, where *** denotes *p* < 0.001, **** denotes *p* < 0.0001, and n.s. denotes non-significant differences. Reprinted with permission from ref. 156. Copyright 2025, American Chemical Society.

FLIM acquisition techniques can be broadly categorised into time-domain and frequency-domain methodologies. Time-domain FLIM typically combines raster scanning of a confocal system with pixel-wise time-correlated single-photon counting (TCSPC), employing pulsed light sources and detectors that precisely measure the arrival times of emitted photons relative to the laser pulses (Fig. 5a). On the other hand, frequency-domain FLIM relies on sinusoidally modulated light sources and phase-shift detection to determine fluorescence decay characteristics from changes in modulation (*M*) and phase (*Φ*) relative to the modulation frequency (Fig. 5b). Time-domain FLIM has been the gold standard for high-resolution lifetime measurements, providing superior contrast, high sensitivity for weakly emitting dyes and the ability to perform label-free imaging.^127^

Classical TCSPC methods record all photons for enhanced sensitivity, which leads to long acquisition times. This is due to the high photon flux generated by most confocal microscopes, which is incompatible with TCSPC systems. As a result, such pile-up distortion causes an underestimation of lifetimes and thus constrains traditional TCSPC systems to long acquisition times. These acquisition times are insufficient for imaging of complex dynamic biological processes that occur at time scales below tens of seconds.^128^ To correct for pile-up distortion, various engineering approaches have been introduced, including ultra-fast TCSPC modules,^129^ single-photon avalanche diode (SPAD) detectors^130^ and field programmable gate array (FPGA)-based electronics,^131^ paving the way for commercially available FLIM microscopes. Other hardware developments to minimise acquisition times have focused on improving the scanning speed and enhancing data acquisition rates. These include single particle tracking FLIM (SPT-FLIM),^132^ light-field tomographic FLIM (LIFT-FLIM)^133^ or high-speed line-scanning fluorescence lifetime microscopes.^134^ These methods improve the imaging speed for FLIM by either reducing the scanning area size or scanning steps required, data readout time or the required photon count per pixel. A discussion of advances in this area is beyond the scope of this review, but such developments address existing speed limitations, allowing time-domain FLIM to serve as a robust method for quantitative biological imaging.

In contrast, frequency-domain FLIM is more suited for high-speed wide-field imaging, with most recent innovations focusing on lifetime unmixing of multiple fluorophores. Traditional frequency-domain techniques suffer from limited multiplexing capabilities as measurements were typically restricted to one or two modulation frequencies at a time.^135^ Newer approaches such as spectrally resolved fluorescent lifetime imaging microscopy (S-FLIM) allow visualisation of more than 5 distinct targets by combining spectral and lifetime information but are subsequently constrained by limited spectral resolution and acquisition speed.^136^ To overcome this, Gratton *et al.* developed Phasor S-FLIM, to integrate true parallel multichannel digital frequency domain (DFD) electronics within a multidimensional phasor approach that enabled simultaneous acquisition of spectral and lifetime information across 32 channels.^137^ This method was successfully applied to MDA-MB231 tumor spheroids for rapid four-color FLIM mapping of lipid droplets (yellow), membranes (red), mitochondria (green) and active mitochondria (blue) using two spectrally similar probes (JC-1 and Nile Red) (Fig. 5c). From both FLIM and average intensity imaging data, Phasor-S-FLIM was able to monitor four key metabolic parameters (*i.e.*, lipid droplet concentration, mitochondrial activity, lipid order, hydrophobicity). Additional instrumentation advances, such as point scanning frequency-domain FLIM with multi-wavelength excitation,^138^ have also improved multiplexing capabilities. Taken together, recent developments position frequency-domain FLIM as a promising alternative for high-speed, multi-target imaging.

### Emerging computational strategies for FLIM data analysis

Regardless of the acquisition method, FLIM generates vast amounts of time-resolved data per image. Conventional approaches for data analysis rely on decay curve fitting, in which the obtained fluorescence decay curve is mathematically fitted to an exponential function based on non-linear least square regressions. FLIMJ, an open-source ImageJ plugin, enables decay curve fitting to be integrated into existing image analysis workflows in ImageJ.^139^ FLIMfit, an open source software, offers an approach to fit data to complex fluorescence decay models, especially for FRET systems.^140^ However, data fitting can be inefficient for bioimaging purposes because the fitting process is computationally intensive, susceptible to noise and dependent on the choice of fitting model.^141,142^ To address these challenges, various computational and AI approaches have been developed to improve speed, accuracy and robustness while reducing photon count requirements.

Phasor analysis provides fast and fit-free analysis for complex multi-component decays, thus becoming the longstanding state-of-the-art for FLIM data interpretation. In the context of FLIM, a phasor is defined as a complex number in which the phase shift (*Φ*) of the emission and the modulation degree (*M*) are used as the angle and amplitude of the phasor, respectively (Fig. 5d).^143^ This can be applied to both time-domain (*via* Fourier transformation) and frequency-domain measurements, simplifying the analysis of FLIM images by transforming complex fluorescence decay profiles into two-dimensional (2D) phasor plots. Pixels of different lifetimes form clusters in the phasor plot, making the phasor approach particularly useful for visualizing lifetime differences between probes of overlapping spectra.^144^ For multiplexed lifetime imaging, the reciprocity principle offers a powerful advantage, where pixels in a specific spatial region of interest can be attributed to points on the phasor plot, therefore identifying individual lifetime populations.^143^ Due to the linear additivity of phasors, the exact co-ordinates of a multi-component phasor are derived from the fractional intensity contributions of its respective components. This enables the decomposition of complex mixtures into individual species, and it is especially useful for applications like FLIM-FRET.^143^

Open-source programs have been developed to improve the accessibility of phasor analysis for lifetime determination and quantitative FLIM imaging. Most of these programs are Python-based and can be integrated with Napari, a Python-based multi-dimensional image viewer. These include Napari-Live FLIM plugin,^145^ the first open-source program designed for real-time FLIM analysis, and napari-FLIM-phasor-plotter,^146^ an open-source plugin for lifetime unmixing based on FLIM phasor analysis. Other Python-based programs include PhasorPy,^147^ a Python-based open-source library for phasor analysis of FLIM data and FLUTE,^148^ a Python GUI that simplifies phasor plot analysis of time-domain FLIM data. While many of these pipelines are not broadly applicable to the average user, they can be adapted to suit the needs of different researchers employing such programs for FLIM research. For example, Schierle *et al.* developed FLIMPA,^149^ a Python-based stand-alone software package for phasor plot analysis. It builds on the capabilities of the Napari-Live FLIM plugin and FLUTE to introduce a versatile application that can be executed in a straightforward manner without any coding expertise, ensuring broader accessibility. These tools have made FLIM data analysis more accessible and flexible, streamlining lifetime imaging workflows and lowering barriers to its adoption in biological research.

Other computational strategies beyond phasor analysis consider the combination of data from multiple sources to improve resolution during FLIM imaging. Faccio *et al.* introduced single-sample image-fusion upsampling (SiSIFUS), a computational super-resolution image fusion pipeline that generates high-resolution lifetime maps from the fusion of high-resolution intensity images (no lifetime information) and low-resolution lifetime images to reduce imaging duration.^150^ Notably, these reconstructed images appeared similar to the “ground truth” (fluorescence lifetime maps obtained using standard deconvolution methods) and possessed enhanced resolution compared to those obtained using bilinear interpolation, a standard upsampling method (Fig. 5e). Computational sensor fusion is another data fusion approach that increases spatial resolution by combining data from SPAD arrays and complementary metal oxide superconductor (CMOS) cameras.^151^ Beyond data fusion, alternative image reconstruction algorithms such as the Bayesian nonparametric framework^152^ and edge-preserving interpolation method (EPIM)^153^ have been utilised to obtain high-pixel FLIM images and unmix lifetimes with subnanosecond resolution from low photon counts. These approaches reduce the high photon requirements typically needed for conventional data analysis methods, making FLIM more robust for real-time dynamic imaging.

AI- and machine learning-based methods have been also developed to overcome low photon counts and long acquisition times. These methods are built on deep learning neural network architectures, and utilise various features of FLIM data (*e.g.*, raw TCSPC data, phasor coordinates, decay curves) to extract spatial lifetime maps. For example, few photon fluorescence lifetime imaging (FPFLI) is a neural network-based method that recognises the spatial correlation of fluorescence lifetimes among neighboring pixels and information obtained from intensity-based images.^154^ UNET-FLIM was trained on synthetic decay curves with known ground truths and developed for fast FLIM data with low photon counts, using a deep learning architecture to determine lifetimes from low photon count data and high background noise levels.^155^ Similarly, FLIMngo accepts raw TCSPC FLIM data and outputs spatial lifetime maps reporting the average lifetime per pixel, using a deep learning model to quantify FLIM data from photon-starved environments.^156^ Schierle *et al.* demonstrated increased performance of FLIMngo against other reported methods by calculating the smallest mean square error for predicted fluorescence lifetime maps (Fig. 5f). On the other hand, SparseFLIM uses a coupled bi-directional propagation network that reconstructs phasor plots from raw sparse FLIM input, based on deep learning and sufficient photon reference data, enriching photon counts in low photon image.^157^ Recently, Leray *et al.* developed PhasorNet, a neural network-based system using phasor plot coordinates, mean and amplitude-weighted lifetimes to analyse FLIM images by estimating bi-exponential decay parameters.^158^ These models take advantage of machine learning to extract lifetime data from photon-starved environments, recovering hidden spatial information from FLIM images. However, because they are machine learning-based, these systems are inherently limited by the samples and datasets that they were trained on. Models such as PhasorNet and FLIMngo were trained on FLIM data acquired with an 80 MHz laser and lifetimes less than 10 ns. These models are thus limited in applicability and would require re-training for fluorophores with longer lifetimes. Furthermore, these models require further validation with broader datasets. Taken together, these frameworks provide the basis for faster FLIM imaging with increased information extraction, paving the way for applications that have traditionally been constrained by low light conditions or high background noise, such as *in vivo* imaging and clinical translation.

Collectively, the advancements in both hardware and software for FLIM address important limitations traditionally associated with time-consuming FLIM experiments. Developments in image acquisition methods will be critical to achieve fast, photon-efficient FLIM imaging of dynamic biological systems while new analysis software will enhance the extraction of meaningful biological information from complex environments. Broader validation of these systems will accelerate the development of more robust methods and the use of FLIM in routine applications. We envision that the integration of AI-driven algorithms with advanced acquisition hardware will play a central role in the translation of FLIM towards more demanding applications.

## Conclusions

Fluorescence lifetime probes hold promise as molecular tools for multicolour live-cell imaging and to obtain semi-quantitative molecular information from subcellular microenvironments. The fluorescence lifetimes of organic fluorophores can be modulated by derivatisation of fluorescent scaffold with chemical groups that inhibit or promote energy transfer and/or charge transfer processes, namely PeT, ICT and TICT. These processes directly affect fluorescence quenching, the stability of excited states as well as non-radiative pathways, thereby altering fluorescence lifetimes. To date, the chemical diversification and conformational rigidification of fluorophores as well as the introduction of responsive elements to specific biological stimuli have been described as effective ways to tailor fluorescence lifetimes. In the long term, we envision that computational approaches will predict electronic distributions and charge-transfer characteristics to accelerate the rational design of fluorophores with optimal properties for FLIM-based imaging, including emerging modalities like FLIM-STED, cryoEM-FLIM and multi-photon FLIM.^159,160^

As FLIM probes provide valuable information about cellular microenvironments, real-time monitoring of variations in fluorescence lifetime allows for biological insights beyond the capabilities of conventional intensity-based fluorescence microscopy. This positions FLIM as an attractive imaging modality for the tracking of organelles and signaling pathways in live cells, biological processes that have implications in pathological states. To date, fluorescence lifetime probes have been chemically engineered to track many different biological processes, from the endo-lysosomal pathway to lipid droplet formation or the mobility of secondary messengers (*e.g.*, Ca^2+^ ions, cAMP). These processes are critical in cellular homeostasis, making fluorescence lifetimes a relevant readout for describing dysregulated pathological states. In recent years, FLIM has been also increasingly employed for the differentiation between healthy and diseased states for cancer as well as neurodegenerative and metabolic disorders, either by tracking endogenous biomolecules (*e.g.*, NADPH) or exogenous fluorophores. We envisage that simultaneous monitoring of endogenous chromophores and targeted fluorescent probes will result in more informative readouts, paving the way for more effective diagnostics and therapy monitoring.

Finally, the integration and translation of fluorescence lifetime probes towards more complex biological systems (*e.g.*, *in vivo* imaging in intact organisms) and clinical applications will require the optimisation of suitable data acquisition and data analysis platforms. Extracting meaningful biological data from FLIM experiments requires balancing photon budgets and measuring fluorescence decay accurately and with high spatiotemporal resolution. Current acquisition strategies typically suffer from long acquisition times and poor accuracy of photon decay. To counter this, some recent developments include methodologies like Phasor S-FLIM, which combines multichannel DFD electronics within a multidimensional phasor approach for simultaneous acquisition of spectral and lifetime information, and Phasor-Net as a phasor-based neural network for fast determination of bi-exponential decay parameters from FLIM images. Furthermore, progress in image acquisition and analysis methods, alongside the integration of AI-driven algorithms, is needed to achieve fast, photon-efficient FLIM imaging of complex biological systems. We anticipate that the design and application of FLIM probes, in combination with advances in data acquisition and analysis methods will further expedite the implementation of FLIM to study multiple biological processes.

## Conflicts of interest

The authors declare no conflicts of interest.

## Data Availability

No primary research results, software or code have been included, and no new data were generated or analysed as part of this review.
